# Vanillin Improves Antioxidant Capacity, Immunity, and Gut Microbiota in Broilers

**DOI:** 10.3390/vetsci13080802

**Published:** 2026-08-14

**Authors:** Baichuan Ma, Jiaxue Wang, Hui Tao, Bing Han, Zhenlong Wang, Yongli Zhang, Jinquan Wang, Xiumin Wang

**Affiliations:** 1 Key Laboratory of Feed Biotechnology, Ministry of Agriculture and Rural Affairs, Institute of Feed Research, Chinese Academy of Agricultural Sciences, Beijing 100081, China; 821012530042@caas.cn (B.M.); hanbing02@caas.cn (B.H.); wangzhenlong02@caas.cn (Z.W.); zhangyongli@caas.cn (Y.Z.); wangjinquan@caas.cn (J.W.); 2 National Nanfan Research Institute (Sanya), Chinese Academy of Agricultural Sciences, Sanya 572024, China; 3 College of Grassland Agriculture, Northwest A&F University, Yangling 712100, China; 19904762097@163.com

**Keywords:** Vanillin, antioxidant activity, anti-inflammatory response, immune function, intestinal microbiota

## Abstract

Wle vanillin is widely used as a flavoring agent in food products, its potential as a non-antibiotic feed additive for broilers remains unexplored. This study primarily investigated the effects of dietary supplementation of vanillin on antioxidant activity, anti-inflammatory response, immune function, and gut microbiota composition in broilers. One-day-old male broilers were randomly assigned to five groups: a basal diet group, a positive control group (basal diet + 100 mg/kg chlortetracycline (CTC)), and three vanillin-supplemented groups (0.1%, 0.2%, or 0.4%) for 42 days. Results showed that vanillin improved antioxidant capacity, with elevated serum superoxide dismutase (SOD) and glutathione peroxidase (GSH-Px) activity and reduced malondialdehyde (MDA) levels. Broilers receiving vanillin supplementation showed lower levels of pro-inflammatory cytokines (interleukin-6 (IL-6), interleukin-1β (IL-1β), and tumor necrosis factor-α (TNF-α)) and higher interleukin-10 (IL-10) expression compared with controls. Additionally, serum immunoglobulin A (IgA) and immunoglobulin M (IgM) levels were also greater in the vanillin group. It also enhanced intestinal health by increasing the duodenal villus height-to-crypt depth ratio, improving intestinal morphology, raising the Firmicutes-to-Bacteroidetes ratio, and promoting *Lactobacillus* proliferation compared with CTC. Notably, growth performance data were exploratory in nature; vanillin was associated with numerical improvements in average daily gain (ADG) and feed conversion (F/G) ratio. These preliminary findings suggest that vanillin requires further investigation as a potential feed additive.

## Simple Summary

This study investigated vanillin as a novel feed additive for broilers. The results showed that vanillin supplementation enhanced antioxidant status and immunity, while alleviating inflammation. Notably, it improved intestinal morphology and favorably modulated the gut microbiota, including increasing the Firmicutes/Bacteroidetes ratio and enriching Lactobacillus populations. These preliminary findings indicate that vanillin is a promising dietary supplementation to promote poultry health.

## 1. Introduction

Antibiotics have served as growth promoters in livestock and poultry production for decades. They can enhance growth performance and production efficiency by inhibiting pathogens, modulating intestinal function, and improving nutrient absorption and feed efficiency, contributing substantively to modern animal production systems [1]. However, their extensive application in animal production has raised global concerns, particularly regarding the emergence and dissemination of antimicrobial resistance (AMR). Prolonged dietary exposure to antibiotics disrupts microbial symbiosis, impairs intestinal immune homeostasis, and weakens host immunity. Moreover, antibiotic residues facilitate the spread of resistant bacteria and resistance genes, which are disseminated into terrestrial and aquatic ecosystems via manure, thereby exacerbating environmental AMR burden [2]. Regulatory authorities worldwide have enacted stringent bans on antimicrobial growth promoters. The European Union (EU) implemented a comprehensive prohibition in 2006, while China enforced a nationwide ban on feed antibiotics in July 2020 (Ministry of Agriculture Announcement No.194) [3]. Consequently, identifying safe, effective, and sustainable alternatives to antibiotics has become an urgent demand for antibiotic-free animal production.

In recent years, significant progress has been made in developing antibiotic alternatives, with promising substitutes including plant extracts, probiotics, prebiotics, antimicrobial peptides (AMPs), etc. [4]. Among these, plant extracts have attracted particular attention due to some advantages, such as abundant source availability, high nutritional value, diverse bioactive properties, low or no toxicity, minimal risk of inducing AMR, and negligible residue accumulation [5]. These attributes make phytogenic additives prime candidates for further evaluation as non-antibiotic feed additives in animal production. Many studies have demonstrated the multifaceted benefits of plant extracts (such as bamboo leaf, ginger root, red osier dogwood, etc.) at 0.1–0.8% in broiler production, including potent antioxidant, anti-inflammatory, antimicrobial, immunomodulatory, and gut microbiota-modulating effects [6,7,8,9,10,11,12,13]. Notably, dietary supplementation with thymol and carvacrol significantly improves antioxidant capacity, as evidenced by increased serum and hepatic superoxide dismutase (SOD) and glutathione peroxidase (GSH-Px) activities, along with reduced malondialdehyde (MDA) levels [14]. Carvacrol essential oil exerts anti-inflammatory effects through dual mechanisms: suppressing pro-inflammatory cytokines and the Toll-like receptors (TLRs)/Nuclear factor-κB (NF-κB) signaling pathway, thereby alleviating lipopolysaccharide (LPS)-induced inflammation [15]. Flavonoids promote immune organ development and modulate immune responses [16], while *Glycyrrhiza* polysaccharides regulate immune overactivation and support host defense mechanisms [17]. Similarly, *Lycium barbarum* polysaccharides help maintain immune homeostasis by regulating anti-inflammatory responses [18]. Furthermore, oregano essential oil and organic acid blends modulate gut microbiota and increase short-chain fatty acid (SCFA) levels. SCFAs play a key role in maintaining intestinal barrier integrity, enhancing systemic anti-inflammatory capacity, promoting nutrient absorption, and improving growth performance in broilers [19].

Vanillin, a bioactive phenolic compound primarily derived from *Vanilla planifolia* beans, is widely applied in the food industry due to its antimicrobial activity [20,21,22,23,24]. Vanillin exhibits potent antioxidant and anti-inflammatory properties with high biosafety [20,25,26,27,28]. Vanillin administration alleviated mastitis in pregnant BALB/c mice [29]. In broilers, supplantation with 0.5 mg/kg vanillin or 0.5% microencapsulated phytochemical blend (containing thymol and vanillin) improved growth performance and antioxidant status, modulated gut microbiome composition, and attenuated systemic inflammation [30,31,32]. Notably, vanillin has been authorized as a feed additive for fattening chickens by the European Commission [33], highlighting its viability for commercial application.

Based on our previous finding that dietary vanillin (60 mg/kg body weight) protected against *Escherichia coli*-induced colitis in mice [34], we hypothesized that dietary vanillin would improve health status and growth efficiency in broilers. To test this hypothesis, we evaluated the effects of vanillin supplementation (0.1–0.4%) on antioxidant capacity, immune function, and cecal microbiota composition. Our results suggest that vanillin is a promising feed additive for sustainable animal production.

## 2. Materials and Methods

### 2.1. Materials

Food-grade synthetic vanillin (purity ≥ 99.50%) was purchased from Jiangxi Yuanshangcao Flavor Co., Ltd. (Jiangxi, China). Commercial ELISA kits for measuring chicken interleukin-6 (IL-6) (catalog number: MM-0521O1), interleukin-1β (IL-1β) (catalog number: MM-36910O1), IL-10 (catalog number: MM-1145O1), and tumor necrosis factor-α (TNF-α) (catalog number: MM-0938O1) were obtained from Jiangsu Meimian Industry Co., Ltd. (Jiangsu, China).

### 2.2. Animals, Diets, and Experimental Design

One-day-old male Kebao broilers (a commercial white-feather broiler strain) were purchased from the Dingjiazhuang Breeding Farm (Beijing Dapa Zhengda Co., Ltd., Beijing, China). All animal experiments and housing conditions were approved by the Experimental Animal Ethics Committee of the Institute of Feed Research of the Chinese Academy of Agricultural Sciences (No. IFR CAAS20241213).

A total of 60 one-day-old Kebao broilers were randomly distributed into five groups; each group was subdivided into four replicate cages (3 birds per cage). The control group received a basal diet, while treatment groups were supplemented with either 0.1%, 0.2%, or 0.4% vanillin. Birds in the positive control group received the basal diet supplemented with 100 mg/kg chlortetracycline (CTC). All diets were formulated to meet or exceed NRC (1994) nutritional requirements across two growth phases: starter (days 1–21) and grower (days 22–42). The detailed dietary formulation and nutritional compositions are provided in Table 1. Upon trial initiation, broilers were housed in multi-tier cages with ad libitum access to feed and water, with twice-daily scheduled feeding. Environmental conditions were strictly controlled, with temperature maintained at 34–36 °C during the first week and gradually reduced by 2–3 °C weekly until reaching a stable temperature of 23 ± 2 °C. Lighting was provided on a 15.5 h light /8.5 h dark cycle. Standard husbandry practices included daily manure removal and ventilation to maintain optimal hygiene and air quality through the experimental period.

### 2.3. Growth Performance Assessment

Broilers were subjected to a 12 h fasting period (with free access to water) prior to body weight measurement on days 21 and 42. Feed intake was recorded weekly through each experimental phase. Growth performance parameters including average daily feed intake (ADFI), average body weight (ABW), and feed conversion ratio were calculated for both the starter (days 1–21) and grower (days 22–42) phases.

### 2.4. Biological Sample Collection

At the end of each experimental phase (days 21 and 42), six healthy broilers per group were randomly selected for blood sample collection. Venous blood (5–6 mL) was aseptically collected from the pterygoid vein using sterile syringes (Beijing zhong Nong si Chen biology science and technology co., ltd., Beijing, China) and transferred to anticoagulant-free tubes. After 2 h of clotting at room temperature, samples were centrifuged at 4000× *g* for 15 min at 4 °C to obtain serum. The serum was aliquoted into sterile tubes and stored at −20 °C. Primary immune organs (such as thymus, spleen, and bursa) and liver were then dissected, weighed, and examined for gross pathology. Liver tissue samples were harvested from standardized lobar locations using sterile instruments, frozen in liquid nitrogen, and stored at −80 °C for further analysis.

The small intestine was carefully segmented into duodenum, jejunum, and ileum. From each bird, a representative 2 cm mid-duodenal segment was collected using the following preservation protocols: frozen in liquid nitrogen for gene expression, and fixed in 4% paraformaldehyde at 4 °C for 24 h for histomorphological analysis.

### 2.5. Serum Biomarker Analysis

Serum biomarkers were analyzed using commercial ELISA kits (Beijing lai bo wo de technology co., ltd., Beijing, China) following manufacturers’ protocols. Immune response was assessed by measuring immunoglobulin levels (such as IgA, IgG, and IgM) using chicken-specific ELISA kits (Beijing lai bo wo de technology co., ltd., Beijing, China). Pro-inflammatory mediators (such as IL-1β, TNF-α, and IL-6) and the anti-inflammatory cytokine IL-10 were measured with chicken-specific ELISA kits. MDA content and the activities of SOD and GSH-Px were quantified using ELISA kits (Beijing lai bo wo de technology co., ltd., Beijing, China). All analyses were performed in three replicates per group.

### 2.6. Duodenum Histopathological Analysis

For histological analysis, duodenal tissues were fixed in 4% paraformaldehyde at 4 °C for 24 h, dehydrated through a graded ethanol series (70–100%), embedded in paraffin, and sectioned at 4 µm using a rotary microtome. The sections were then dewaxed in xylene, rehydrated through a graded alcohol series, and stained with hematoxylin and eosin (H&E) using a standard protocol, including hematoxylin differentiation in 1% acid alcohol, bluing in 0.2% ammonia waterand final dehydration prior to mounting with neutral balsam. Histomorphological evaluation was performed using a fluorescence microscopy (Nikon, Tokyo, Japan, Eclipse Ci-L) equipped with a digital imaging system. Briefly, three non-consecutive sections (5 µm) were prepared per bird; 10 intact, well-oriented villi and their corresponding crypts were randomly selected and measured, resulting in 30 villi and 30 crypts evaluated per bird. Villus height, crypt depth, and the V/C (Villus height/Crypt depth) ratio were measured by an observer blinded to the treatment groups to minimize bias [35].

Quantitative morphometric analysis was performed to assess key intestinal parameters, including crypt depth (CD), intestinal wall thickness (IWT), villus height (VH), and the villus height-to-crypt depth ratio. A comprehensive pathological scoring system was used to evaluate mucosal integrity (epithelial continuity and villus structure), glandular architecture (crypt morphology and dilation), cellular components (goblet cell density and inflammatory infiltration), and pathological changes (such as edema, hemorrhage, crypt abscess formation, and epithelial dysplasia).

### 2.7. 16S rRNA Sequencing of Cecal Microbiota

Cecal content samples (≥2 g per biological replicate) were aseptically collected and immediately frozen in liquid nitrogen prior to storage at −80 °C for subsequent microbial analysis. Total genomic DNA was extracted using the TIANamp Stool DNA Kit (TIANGEN, Beijing, China) following the manufacturer’s protocol, including mechanical lysis and proteinase K digestion to ensure maximal cellular disruption and DNA yield. DNA concentration and purity were determined using the Qubit dsDNA HS Assay Kit (Thermo Fisher Scientific, Waltham, MA, USA) with fluorescence measurement. All DNA extracts were aliquoted and stored at −80 °C until PCR amplification.

The V3–V4 hypervariable regions of bacterial 16S rRNA genes were amplified using universal primers at 20 s and 72 °C for 30 s. The reaction was 338F (5′-CCTACGGGNGGCWGCAG-3′) and 806R (5′-GACTACHVGGGTATCTAATCC-3′). PCR amplification was performed under the following conditions with minor modifications: initial denaturation at 94 °C for 3 min, followed by 20 cycles of denaturation (94 °C, 30 s), annealing (55 °C, 20 s), and extension (72 °C, 30 s). An additional 20 cycles were then conducted with modified conditions: 94 °C for 20 s, 55 °Completed with a final extension at 72 °C for 5 min [36,37]. Sterile PBS or nuclease-free water was used as the blank or negative control [38]. PCR products were purified using the QIAquick Gel Extraction Kit (Qiagen, Venlo, The Netherlands) and quantified using the Quant-iT PicoGreen dsDNA Assay Kit (Thermo Fisher Scientific, Waltham, MA, USA). Purified amplicons were then submitted to paired-end sequencing on the Illumina platform (Sangon Biotech, Shanghai, China).

The V3–V4 region of 16S rRNA was sequenced on an Illumina platform. Raw paired-end reads were preprocessed as follows: primer-adapter sequences of Read1/Read2 were removed via cutadapt; overlapping PE reads were merged using PEAR; sequences were demultiplexed by barcodes and orientation-corrected. Quality filtering was performed in PRINSEQ with a 10 bp sliding window (truncating tails where average Q < 20). Reads with ambiguous N bases, short fragments and low-complexity sequences were discarded to yield clean reads.

After filtering, samples contained 67,096–116,620 clean reads (mean ≈ 94,977 reads per sample). Rarefaction curves plateaued, indicating adequate coverage; all samples were rarefied to 67,096 reads (minimum depth) prior to diversity analysis. Taxonomic assignment of OTU representative sequences was conducted via the RDP Classifier with the Naïve Bayesian algorithm against the RDP reference database, and community composition was quantified across seven taxonomic levels: domain, phylum, class, order, family, genus, and species.

### 2.8. Statistical Analysis

Microbial community analysis was performed using QIIME and R (version 3.5.1). Alpha diversity (Shannon index) and bacterial abundance were compared across groups using Kruskal–Wallis tests, followed by pairwise Mann–Whitney U tests with false discovery rate (FDR) correction. Beta diversity was assessed using UniFrac distance metrics and visualized via principal coordinate analysis (PCoA). To identify differentially abundant taxa, we performed STAMP (version 2.1.3) and LEfSe (version 1.1.0) analyses. The LEfSe analysis was performed using a linear discriminant analysis (LDA) score threshold of > 2.0 to identify taxa with significant differential abundance among groups. Microbial co-occurrence networks were constructed using SparCC (version 1.1.0), which calculates correlation coefficients and *p*-values between operational taxonomic units (OTUs).

All statistical analyses were performed in GraphPad Prism (version 9.0). Data are presented as mean ± standard deviation (SD). Before parametric statistical analyses, data normality was assessed using the Shapiro–Wilk test. Group differences were evaluated using one-way ANOVA with post hoc least significant difference (LSD) tests, and statistical significance was set as *p*-value < 0.05.

## 3. Results

### 3.1. Effects of Vanillin on Growth Performance and Immune Organs in Broilers

Dietary vanillin appeared to increase final body weight at both day 21 and 42 (*p* < 0.05). In contrast, CTC supplementation had no significant effect on body weight over the same period (Table 2). Vanillin also improved average daily gain (ADG) and reduced the feed-to-gain (F/G) ratio across the experimental period. The observations suggest a possible growth-promoting potential of vanillin.

As shown in Table 3, dietary supplementation with 0.2% and 0.4% vanillin significantly decreased relative pancreatic weight in 21-day-old broilers compared with the control group (*p* < 0.05), while 0.1% vanillin increased relative pancreatic weight to a level higher than that in the CTC group. Additionally, 0.4% vanillin reduced relative spleen weight, whereas 0.1% vanillin increased the relative weights of both the bursa and spleen (*p* < 0.05). By day 42, bursa weight declined across all groups compared with day-21 values. Notably, CTC and all vanillin doses decreased relative bursa weight compared with the control group (*p* < 0.05).

### 3.2. Effects of Vanillin on Serum Anti-Inflammatory Factors in Broilers

**Figure 1:**
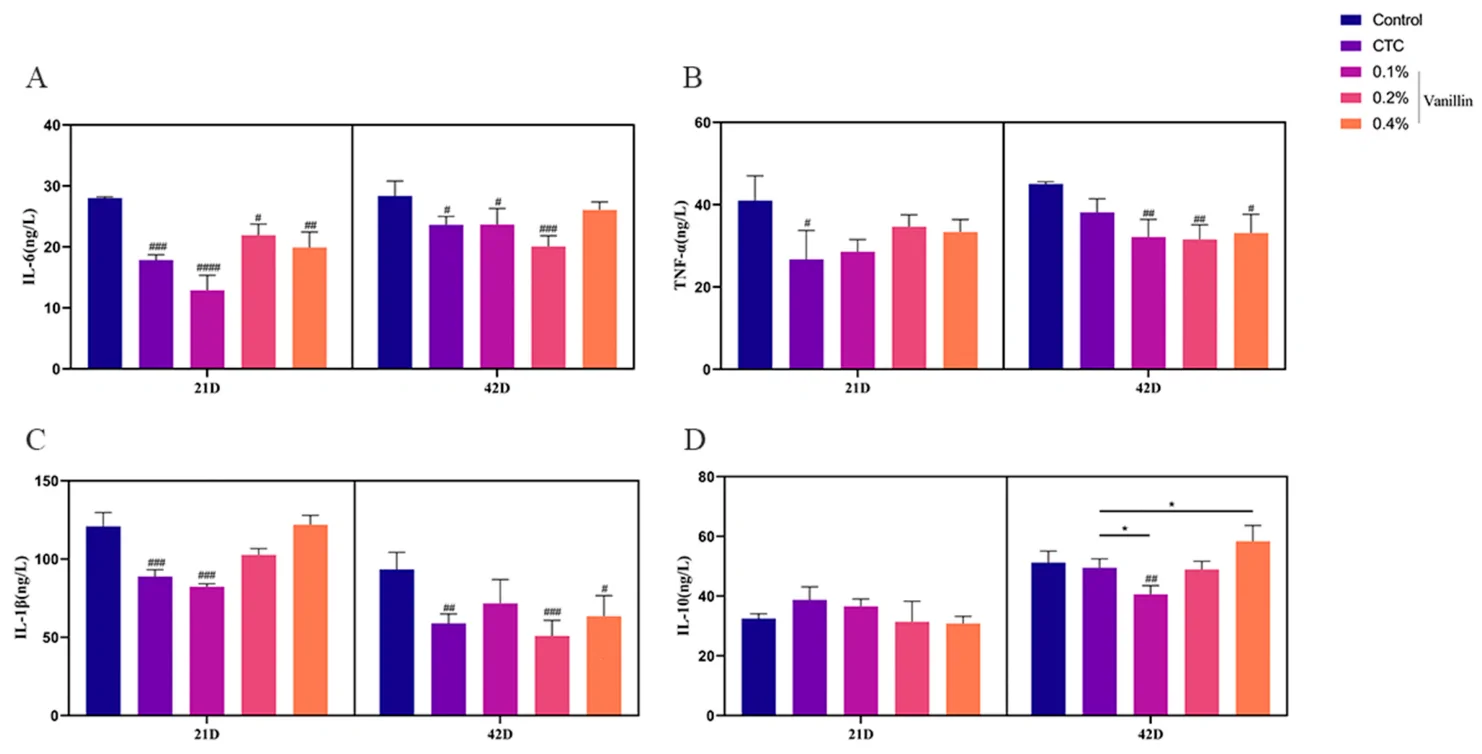
Effect of Vanillin on Serum Inflammatory Factors in Broilers at 21 and 42 Days of Age. (**A**) Effect on IL-6 expression. (**B**) Effect on IL-1β expression. (**C**) Effect on TNF-α expression. (**D**) Effect on IL-10 expression. Blood samples were obtained on days 21 and 42, and cytokine concentrations were quantified using ELISA kits. The data are expressed as the mean ± SD (*n* = 6). Significantly different from the CTC group (* *p* < 0.05). Significantly different from the control (# *p* < 0.05, ## *p* < 0.01, ### *p* < 0.001, #### *p* < 0.0001).

As shown in Figure 1, dietary vanillin reduced serum IL-6 levels compared with the control group at day 21 (*p* < 0.05), with 0.1% vanillin showing the strongest suppression. Notably, 0.1% vanillin also decreased IL-1β and TNF-α (*p* < 0.001) while increasing the anti-inflammatory cytokine IL-10. By day 42, all vanillin doses significantly modulated both pro- and anti-inflammatory cytokines. Specifically, 0.1% vanillin reduced serum IL-6 and TNF-α levels at this time point. Compared with day-21 values, serum IL-10 levels increased across all groups by day 42, with vanillin supplementation showing a greater elevation than both the control and CTC groups (*p* < 0.05). These findings indicate that dietary vanillin exerts anti-inflammatory effects in broilers.

### 3.3. Effects of Vanillin on Antioxidant Factors in Broilers

**Figure 2:**
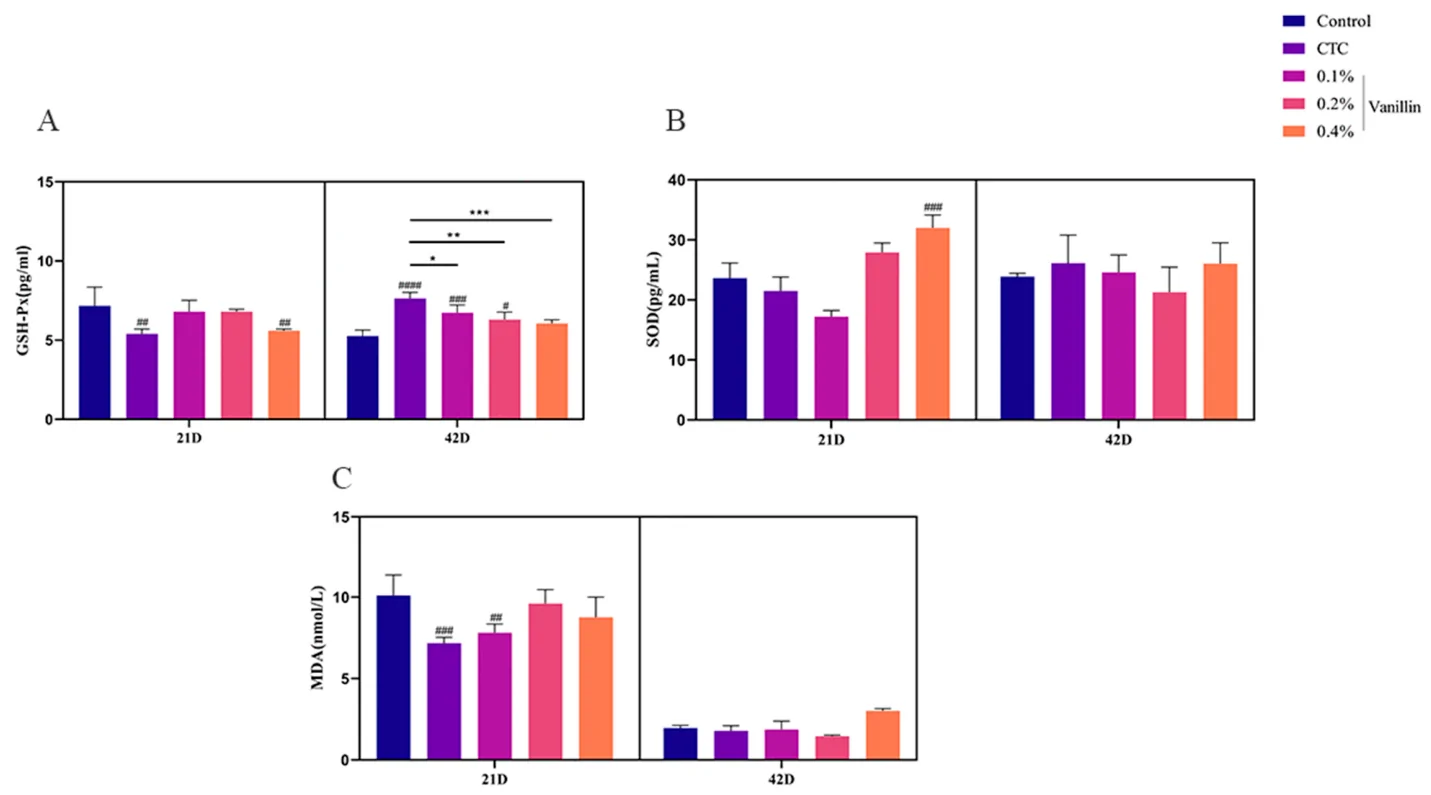
Effects of Vanillin on Serum Antioxidant Factors in Broilers at 21 and 42 Days of Age. (**A**) Effect on GSH-Px activity. (**B**) Effect on SOD activity. (**C**) Effect on MDA level. Blood samples were obtained on days 21 and 42, and antioxidant enzyme activities and MDA levels were determined using ELISA kits. The data are expressed by the mean ± SD (*n* = 6). Significantly different from the CTC group (* *p* < 0.05, ** *p* < 0.01, *** *p* < 0.001). Significantly different from the control (# *p* < 0.05, ## *p* < 0.01, ### *p* < 0.001, #### *p* < 0.0001).

Dietary vanillin modulated serum antioxidant enzyme profiles. At day 21, CTC significantly reduced GSH-Px activity compared with the control group, whereas vanillin groups showed no significant difference from the control (Figure 2A). Notably, 0.4% vanillin markedly elevated SOD activity by 35% relative to the control (*p* < 0.001) (Figure 2B), and 0.1% vanillin decreased MDA levels by 29% compared with the control (*p* < 0.05) (Figure 2C). By day 42, all vanillin doses increased GSH-Px and SOD activities compared with the control (*p* < 0.001). The results indicate that dietary vanillin may enhance antioxidant status in broilers.

### 3.4. Immunomodulatory Effects of Vanillin in Broilers

**Figure 3:**
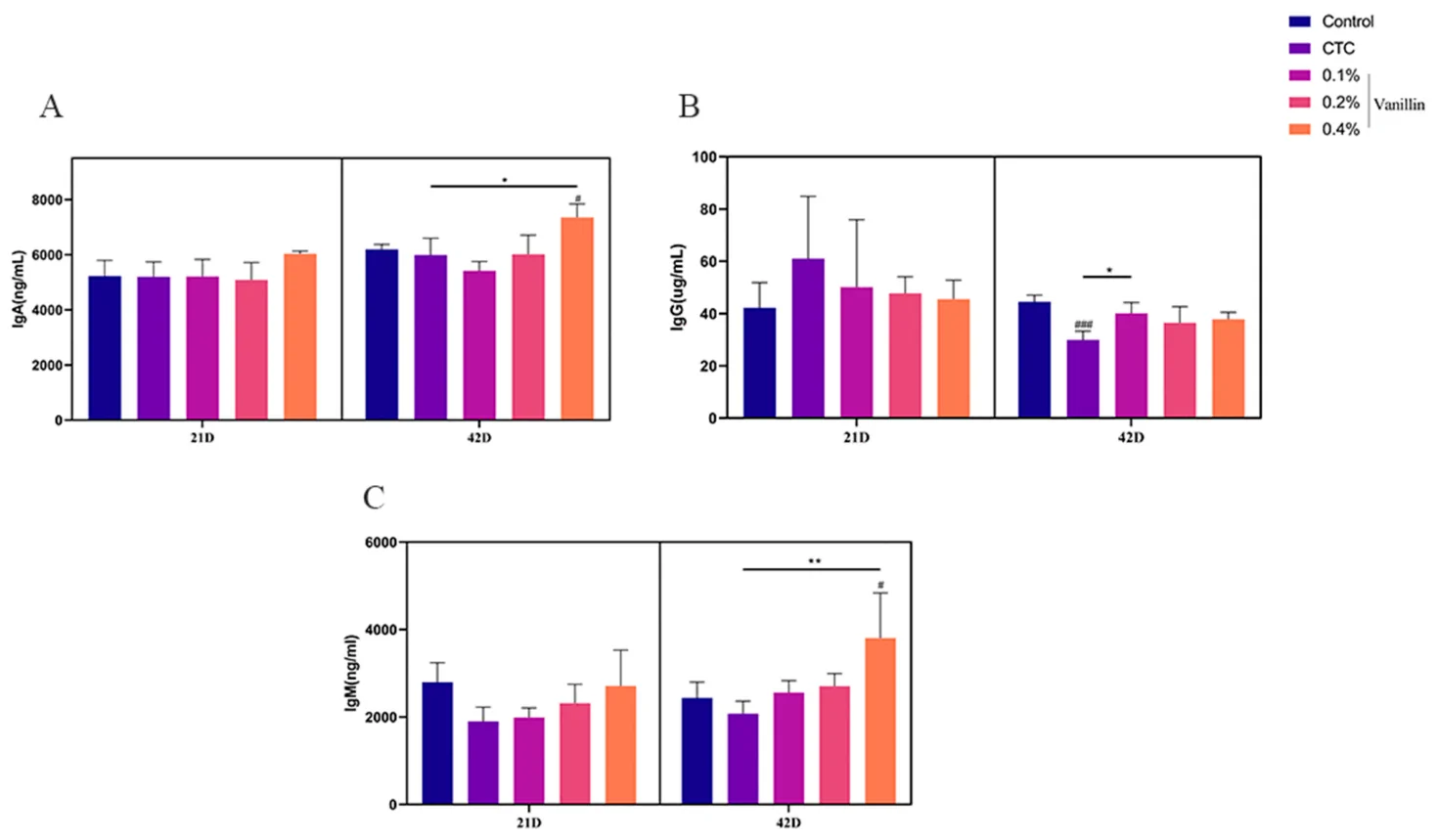
Immunomodulatory Effect of Vanillin in Broilers at 21 and 42 Days of Age. (**A**) Effect on IgA expression. (**B**) Effect on IgG expression. (**C**) Effect on IgM expression. Blood samples were obtained on days 21 and 42, and immunoglobulin levels were measured using ELISA kits. The data are expressed by the mean ± SD (*n* = 6). Significantly different from the CTC group (* *p* < 0.05, ** *p* < 0.01). Significantly different from the control (# *p* < 0.05, ### *p* < 0.001).

At day 21, broilers fed 0.4% vanillin increased serum IgA levels compared with both the control and CTC groups (*p* < 0.05) (Figure 3). By day 42, the 0.4% vanillin treatment increased IgA and IgM concentrations by 23% and 83%, respectively, relative to the CTC group (*p* < 0.05). These results suggest that dietary vanillin may enhance immunity in broilers.

### 3.5. Effects of Vanillin on Duodenal Morphology in Broilers

**Figure 4:**
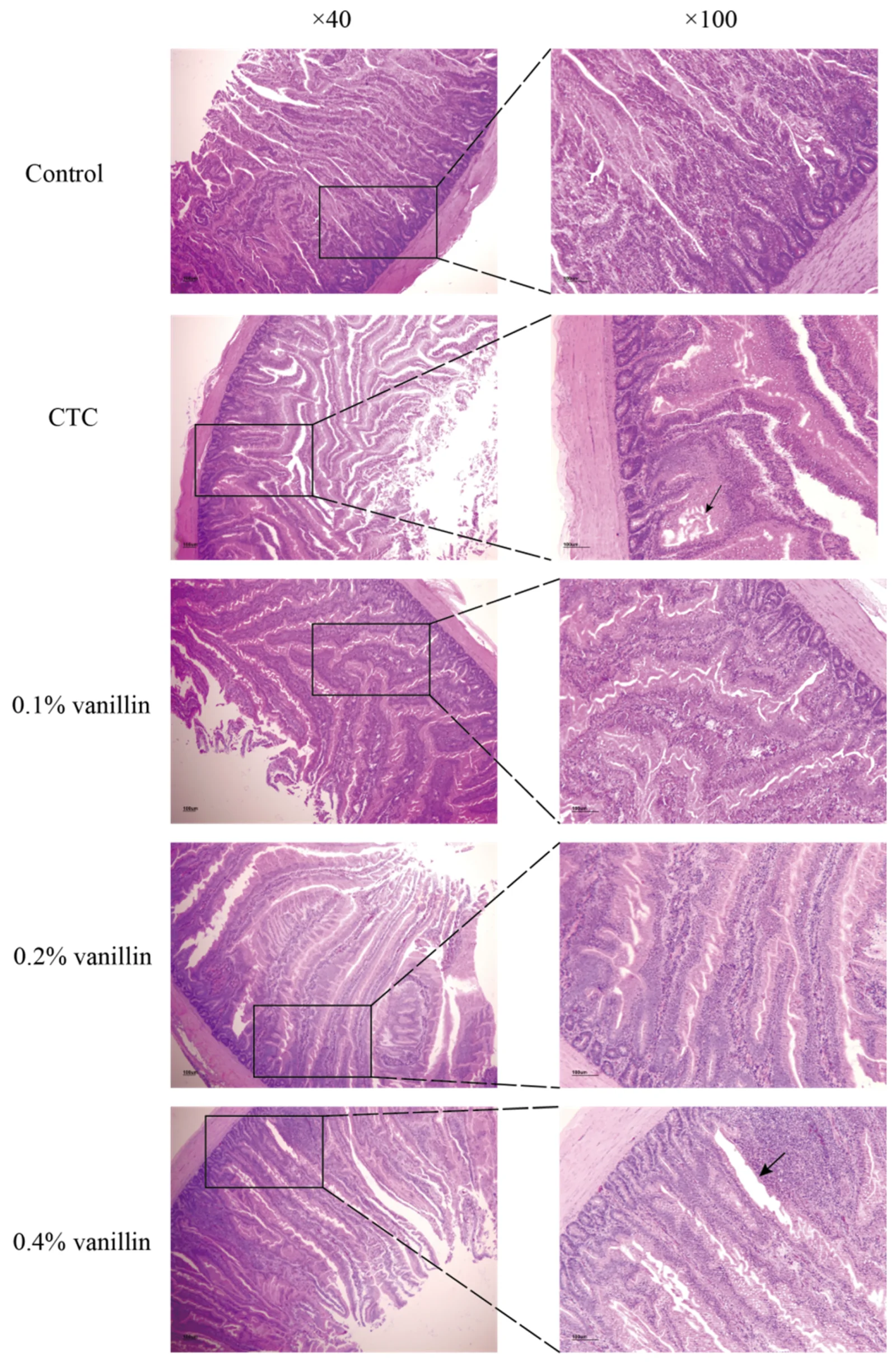
Effect of Vanillin on Duodenal Morphology in Broilers at 42 d of Age. Tissues were fixed (4% PFA), paraffin-embedded, and cut into 4 µm sections. After H&E staining and dehydration, images were captured using a light microscope for morphological assessment.

Dietary supplementation with 0.1% and 0.4% vanillin reduced duodenal crypt depth (*p* < 0.05) (Table 4). Notably, 0.1% vanillin increased the V/C ratio by 53% (*p* < 0.05) compared with the CTC group, suggesting improved intestinal absorptive capacity. Histopathological analysis revealed epithelial and glandular structural damage in the CTC group, whereas the 0.1% vanillin group maintained an intact mucosal surface characterized by clearly visible folds and normal glandular morphology without detectable lesions (Figure 4). In contrast, broilers fed 0.4% vanillin exhibited mild epithelial disruption. Thus, vanillin may optimize intestinal morphology.

### 3.6. Effects of Vanillin on Cecal Microbiota in Broilers

**Figure 5:**
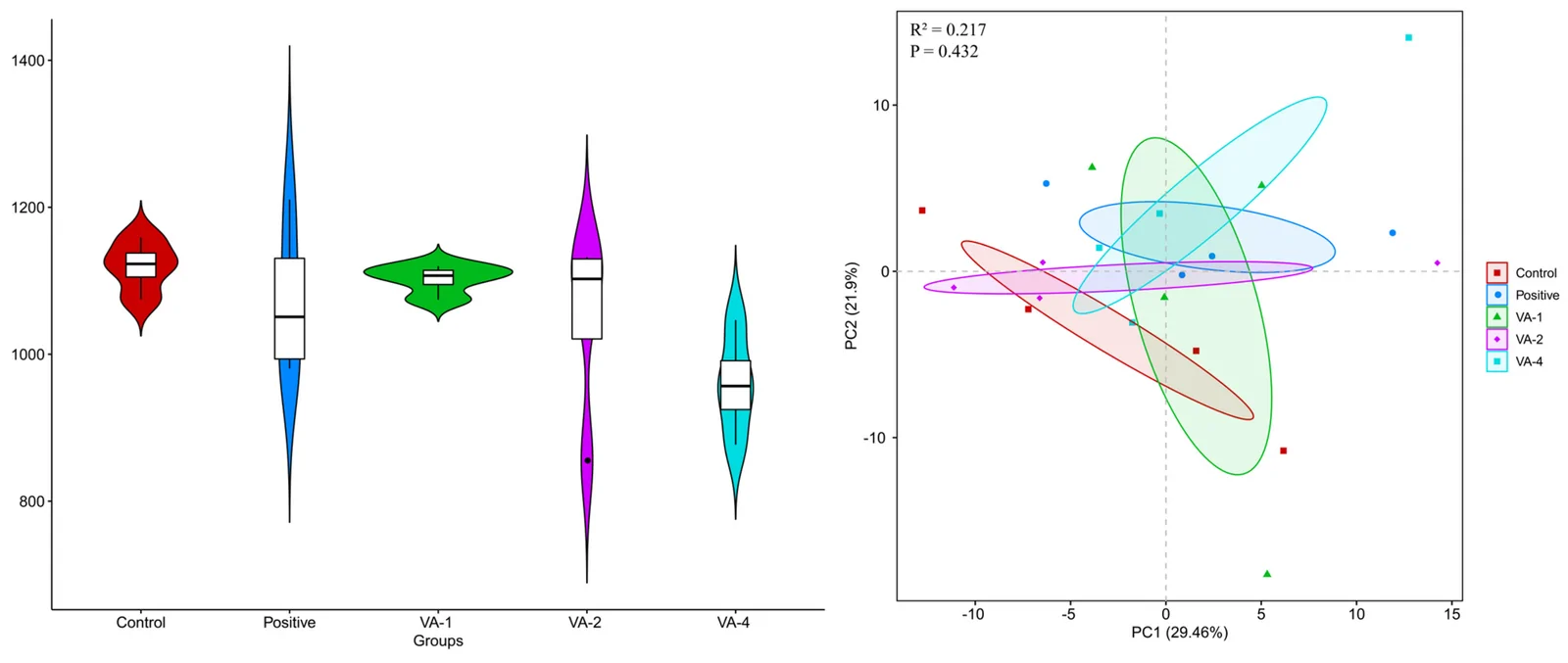
Effects of Vanillin on Cecal Microbiota in Broilers. (**left**) Alpha diversity index. VA-1: 0.1% vanillin was added to the diet; VA-2: 0.2% vanillin was added to the diet; VA-4: 0.4% vanillin was added to the diet. (**right**) PCA at genus level. Cecal contents were collected at 42 days of age; genomic DNA was extracted, and the V3–V4 regions of the 16S rRNA gene were amplified and sequenced on an Illumina platform.

Microbial diversity analysis revealed distinct clustering patterns among treatment groups (Figure 5A). Principal component analysis (PCA) suggested no major restructuring of the cecal microbiota (Figure 5B). Notably, the CTC-treated group formed a separate cluster distinct from the control, whereas all vanillin-supplemented groups (0.1% VA-1, 0.2% VA-2, and 0.4% VA-4) clustered closely with the control, exhibiting substantial overlap in community distribution. These findings indicate that vanillin helps maintain cecal microbial homeostasis relative to CTC and support its potential as a non-antibiotic feed additive for broilers.

**Figure 6:**
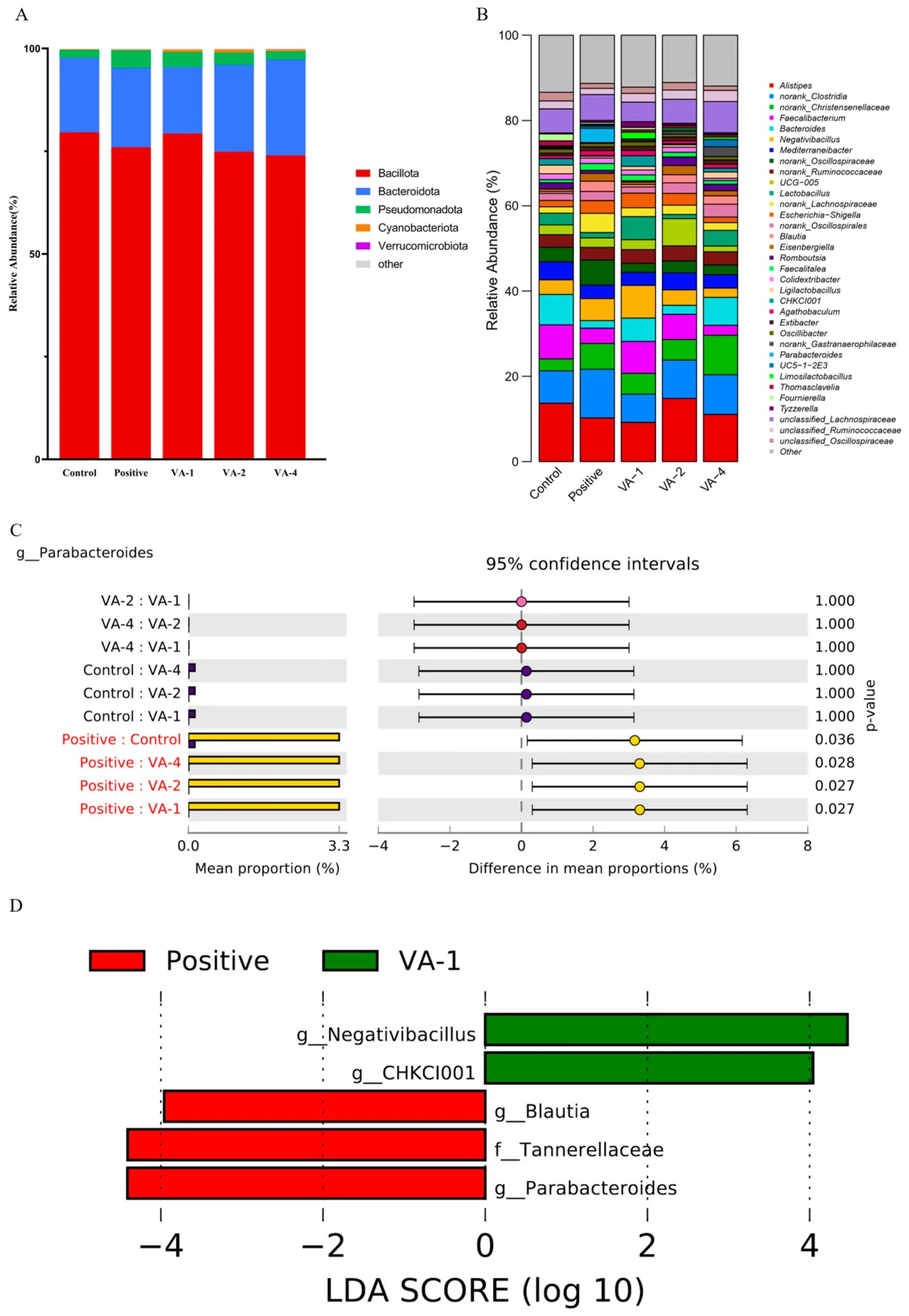
Effects of Vanillin on Cecal Microbiota Composition of Broilers. (**A**) Phylum-level microbial community structure; (**B**) Genus-level taxonomic distribution; (**C**) Differential abundance analysis of Parabacteroides at genus level; (**D**) LEfSe analysis histogram of bacterial taxa among treatment groups. Cecal contents were collected at 42 days of age; genomic DNA was extracted, and the V3–V4 regions of the 16S rRNA gene were amplified and sequenced on an Illumina platform.

No significant difference in taxonomic composition was detected at the phylum level among groups (Figure 6A). The cecal microbiota was dominated by five phyla: Bacillota, Bacteroidota, Pseudomonadota, Cyanobacteriota, and Verrucomicrobiota. The control group was characterized by Bacillota (79.59%), Bacteroidota (18.12%), Pseudomonadota (1.91%), and Cyanobacteriota (0.16%), with a similar distribution in the CTC group (Bacillota: 76.00%; Bacteroidota: 19.29%; Pseudomonadota: 3.67%; Cyanobacteriota: 0.57%). Vanillin modulated cecal microbiota composition (Figure 6A). The VA-1 group (0.1% vanillin) maintained a profile comparable to the control (Bacillota: 79.32%; Bacteroidota: 16.15%), while higher doses increased Pseudomonadota abundance (VA-2: 21.13%; VA-4: 23.32%) concomitantly with reduced Bacillota levels (VA-2: 74.90%; VA-4: 73.99%). This shift was reflected in the Firmicutes/Bacteroidota ratio, which declined dose-dependently: 4.91 (VA-1), 3.55 (VA-2), and 3.17 (VA-4), compared with 4.39 (Control) and 3.94 (CTC group). Notably, CTC significantly increased Pseudomonadota, whereas 0.1% vanillin selectively enriched both Bacillota and Bacteroidota while maintaining microbial equilibrium. The findings suggest that vanillin may modulate cecal microbiota composition, supporting its utility as a dietary intervention for gut health management in broilers.

Genus-level analysis revealed 10 dominant microbial taxa, including *Alistipes*, unclassified_*Clostridia*, unclassified_*Christensenellaceae*, *Faecalibacterium*, *Bacteroides*, *Negativibacillus*, *Mediterraneibacter*, unclassified_*Oscillospiraceae*, unclassified_*Ruminococcaceae*, and *UCG-005* (Figure 6B). Notably, the VA-1 group (0.1% vanillin) exhibited significant enrichment of lactic acid bacteria compared with other treatment groups. While the CTC group showed marked alterations in *Parabacteroides* and *Blautia* abundance (*p* < 0.05) (Figure 6C), VA-1 was characterized by a distinct probiotic profile, with elevated levels of beneficial genera *Negativibacillus* and *CHKCI001* (*p* < 0.05) (Figure 6D). The result indicates that dietary vanillin may promote probiotic colonization.

## 4. Discussion

This study provides a preliminary assessment of vanillin as a dietary feed additive in broiler production. We aimed to determine whether vanillin could support physiological performance by modulating antioxidant status, immune function, and gut microbiota composition.

The development and functional integrity of avian immune organs, particularly the thymus, spleen, and bursa, serve as fundamental indicators of immunological competence in poultry, mediating both cellular and humoral immunity while maintaining immune homeostasis [39]. Probiotic interventions, including *Lactobacillus casei* KL1 [40] and *Lactobacillus plantarum* [41], have been shown to enhance immune organ development, as reflected by relative organ weights. The pancreas likewise contributes critically to broiler health by supporting digestive enzyme secretion and intestinal mucosal immunity [42,43,44]. In this study, dietary vanillin was associated with numerically higher relative weights of the pancreas (16%), bursa (78%), and spleen (38%) in 21-day-old broilers compared with the control (Table 3). By day 42, these differences largely attenuated, coinciding with natural physiological involution and the dilution effect of increased body weight gain [45]. It should be noted that physiological involution occurs universally in aging broilers; thus, the observed differences likely represent modulatory effects rather than alterations of involution itself. The preliminary findings require further investigation into vanillin’s potential as a non-antibiotic dietary supplement.

The cellular antioxidant defense system, primarily mediated by SOD and GSH-Px, maintains redox homeostasis and prevents oxidative damage. SOD converts superoxide anions (O_2_^−^) to hydrogen peroxide (H_2_O_2_), which GSH-Px and catalase (CAT) subsequently reduce to water [46]. Selenium-dependent GSH-Px additionally protects cell membrane integrity by reducing lipid hydroperoxides and preventing polyunsaturated fatty acid (PUFA) peroxidation [47]. When oxidative stress exceeds endogenous defense capacity, O_2_^−^ initiates lipid peroxidation, producing MDA as a stable biomarker of oxidative damage [48,49]. In this study, vanillin supplementation modulated antioxidant status in a time-dependent manner, increasing SOD activity by 35% at day 21 (*p* < 0.05) and GSH-Px activity by 28% at day 42 (*p* < 0.05), while reducing MDA concentrations by 29% compared with the control (Figure 2). The results support vanillin as an effective dietary antioxidant for poultry production, although the mechanisms underlying these effects remain to be fully elucidated.

Avian immunoglobulins–IgG, IgM, IgA, and IgY–synergistically protect against pathogens [50], with IgG and IgM primarily mediating systemic immunity and IgA safeguarding mucosal surfaces. In this study, vanillin increased IgA and IgM levels by 19% and 56%, respectively, while moderately reducing IgG levels by 11% (Figure 3), which indicates a strategic reallocation of immune resources toward mucosal defense–the first line of protection against enteric pathogens–accompanied by attenuated systemic IgG production. This finding is consistent with previous reports of the immunomodulatory effects induced by *L. plantarum* [41] and suggests that vanillin may promote a balanced immune response in broilers.

The inflammatory response in poultry is regulated by the dynamic balance between pro- and anti-inflammatory cytokines; its disruption underlies various pathological conditions. Under homeostatic conditions, precise regulation maintains alertness while preventing excessive activation. However, pathogenic challenges trigger exaggerated pro-inflammatory responses characterized by the overexpression of IL-1β, IL-6, and TNF-α–the latter serving as a key initiator of inflammatory cascades through NF-κB activation [51]. Such inflammatory dysregulation may be associated with intestinal barrier dysfunction through disruption of tight junction proteins [52]. In our study, vanillin supplementation reduced serum levels of pro-inflammatory cytokines (IL-6, IL-1β, and TNF-α) in 42-day-old broilers (*p* < 0.05) while upregulating the anti-inflammatory cytokine IL-10 (Figure 1). These results are consistent with previous reports on phytogenic compounds (such as plant essential oils and organic acids), which exert anti-inflammatory effects through modulation of the NF-κB and MAPK pathways [53,54] (Zhe et al., 2008). However, the mechanisms underlying vanillin-mediated immunomodulation remain to be elucidated in future studies.

The structural integrity of the avian small intestine represents a key determinant of physiological performance, coordinating nutrient absorption, immune regulation, and microbial homeostasis. Histomorphological parameters, particularly the V/C ratio, serve as a reliable biomarker of intestinal maturity and absorptive efficiency [55]. In this study, dietary 0.1% vanillin improved duodenal morphology, increasing the V/C ratio by 53% compared with the control, superior to the CTC group (Table 4). The low-dose improvement may tentatively reflect mild stimulation of intestinal epithelial turnover, while higher doses may evoke compensatory responses consistent with a hormesis model [56,57,58]. Such biphasic responses are commonly reported for bioactive feed additives, wherein efficacy peaks at an optimal dose and diminishes at higher concentrations due to metabolic overload–a hormetic pattern previously observed with berberine [59] and other alkaloid-based compounds [60,61]. These interpretations remain speculative and require further investigation. The observed pattern is consistent with previous studies on phytogenic growth promoters [55,62,63], suggesting vanillin’s potential as a modulator of intestinal morphology.

Dietary vanillin enriched beneficial taxa at both the phylum and family levels, including Bacillota, Bacteroidota, and the Lactobacillaceae family (Figure 6), consistent with the findings of Deryabin et al. [27], who demonstrated that vanillin enriches taxa linked to improved growth performance. At the genus level, vanillin increased the relative abundance of *Lactobacillus*, *Negativibacillus*, and *CHKCI001*—three functional taxa associated with gut health and nutrient harvest. *Lactobacillus* is a well-established probiotic that reinforces intestinal barrier integrity and stimulates digestive enzyme activity across diverse animal species [58,64,65]. *Negativibacillus* produces butyrate that elevates cecal palmitic and stearic acids to promote epithelial health [66,67]. Meanwhile, *CHKCI001* (Clostridiaceae) contributes via distinct carbohydrate fermentation pathways, converting indigestible polysaccharides into SCFAs and correlating with improved feed conversion and growth performance in poultry [68,69]. The co-enrichment of these genera suggests that vanillin may remodel the cecal microbiome toward a profile characterized by reinforced barrier function and enhanced energy harvest.

## 5. Conclusions

This preliminary study suggests that dietary vanillin may enhance antioxidant capacity, modulate immune responses, improve intestinal morphology, and reshape gut microbiota composition in broilers. Vanillin was also associated with numerical improvements in growth performance, although these data were exploratory in nature, limiting statistical interpretation. Therefore, while vanillin shows potential as a feed additive, these findings require further validation in dedicated growth trials to confirm its efficacy and optimize dosage for sustainable poultry production.

## Figures and Tables

**Table 1:** Composition and Nutrient Content of Experimental Diets.

Ingredients (g/kg) ^1^	0–21 d	21–42 d
Corn	53.19	54.30
Soybean meal	34.75	30.88
Corn gluten meal	3.56	4.85
Soybean oil	3.82	6.16
Dicalcium phosphate	2.18	1.72
Limestone	0.91	0.68
DL-Methionine	0.31	0.27
L-Lysine	0.37	0.30
Sodium bicarbonate	0.15	0.15
NaCl	0.15	0.15
Choline Cloride	0.10	0.10
L-Threonine	0.11	0.09
L-Valine	0.09	0.04
L-Arg	0.05	0.05
L-Ile	0.02	0.02
Premix1	0.22	0.22
Antioxidant	0.02	0.02
Total	100.00	100.00
**Nutrient level ^2^**		
AMEn Poultry, kcal/kg	3050	3250
CP, %	23.00	22
Ca, %	0.95	0.75
P, %	0.76	0.68
SID Lys Poultry, %	1.28	1.15
SID Met Poultry, %	0.63	0.59
SID Thr Poultry, %	0.81	0.76

^1^ Composition and nutrient content of experimental diets. ^2^ Premix supplied per kg of diet: VA 12,000 IU, VD3 2500 IU, VE 15 IU, VK3 2.65 mg, VB1 2 mg, VB2 6 mg, VB12 0.025 mg, biotin 0.0325 mg, folic acid 1.25 mg, Ca-pantothenate 12 mg, niacin 50 mg, Cu 8 mg, Zn 75 mg, Fe 80 mg, Mn 100 mg, Se 0.15 mg, I 0.35 mg. AMEn: N-corrected apparent metabolizable energy. CP: crude protein. SID: standardized ileal digestibility.

**Table 2:** Effects of Dietary Vanillin Supplementation on Growth Performance of Broilers.

Items	Control	CTC	0.1% Vanillin	0.2% Vanillin	0.4% Vanillin
1 d ABW (g)	40.78	40.78	40.78	40.78	40.78
21 d ABW (g)	902.38 ± 42.40 ^a^	926.12 ± 58.2 ^a^	906.83 ± 68.62 ^a^	947.88 ± 29.24 ^b^	959.33 ± 50.80 ^c^
42 d ABW (g)	3022.50 ± 34.64 ^a^	2997.50 ± 41.13 ^a^	3132.50 ± 99.12 ^ab^	3195.00 ± 72.34 ^ab^	3290.75 ± 137.34 ^b^
1 to 21 d					
ADFI (g/d/bird)	53.94	55.69	58.07	55.39	55.40
ADG (g/d/bird)	41.03	42.16	39.26	43.20	41.53
F/G (g/g)	1.31	1.32	1.48	1.28	1.33
21 to 42 d					
ADFI (g/d/bird)	150.68	146.53	149.04	146.59	138.30
ADG (g/d/bird)	100.96	98.63	107.96	107.01	113.23
F/G (g/g)	1.49	1.49	1.38	1.37	1.22
1 to 42 d					
ADFI (g/d/bird)	102.31	101.11	103.56	101	96.85
ADG (g/d/bird)	71.00	70.40	73.61	75.10	77.38
F/G (g/g)	1.44	1.44	1.41	1.34	1.25

Control: blank control; CTC: chlortetracycline. ABW: average body weight; ADFI: average daily feed intake; ADG: average daily gain; F/G ratio: feed-to-gain ratio. ABW: values are mean ± SD (*n* = 4 pens); different lowercase letters indicate significant differences (*p* < 0.05). ADG, ADFI, FCR: values are single pen-level aggregates derived from initial and final measurements; SD is not reported. The parameters were collected as exploratory data and are presented descriptively.

**Table 3:** Effects of Dietary Vanillin Supplementation on Relative Organ Weights of Broilers at 21 and 42 Days of Age.

Items (g/kg)	Control	CTC	0.1% Vanillin	0.2% Vanillin	0.4% Vanillin
21 d					
Pancreas	3.84 ± 0.34 ^b^	3.88 ± 0.24 ^b^	4.47 ± 0.37 ^b^	3.30 ± 0.08 ^a^	3.43 ± 0.33 ^a^
Bursa of *Fabricius*	2.51 ± 0.31 ^a^	2.47 ± 0.21 ^a^	4.48 ± 0.31 ^b^	2.28 ± 0.20 ^a^	2.72 ± 0.07 ^a^
Spleen	1.12 ± 0.04 ^b^	1.06 ± 0.12 ^b^	1.54 ± 0.06 ^c^	1.04 ± 0.03 ^b^	0.86 ± 0.02 ^a^
Liver	26.83 ± 1.30	27.16 ± 1.34	26.02 ± 0.88	26.87 ± 0.35	24.68 ± 1.29
42 d					
Pancreas	1.74 ± 0.10	1.84 ± 0.07	1.57 ± 0.26	1.62 ± 0.10	1.71 ± 0.22
Bursa of *Fabricius*	0.77 ± 0.08 ^c^	0.59 ± 0.05 ^ab^	0.55 ± 0.07 ^ab^	0.67 ± 0.03 ^bc^	0.49 ± 0.09 ^a^
Spleen	1.07 ± 0.16	0.93 ± 0.02	1.26 ± 0.32	0.90 ± 0.06	1.24 ± 0.19
Liver	17.50 ± 0.78	18.78 ± 0.70	18.37 ± 1.05	17.46 ± 0.84	17.90 ± 0.71

Control: blank control; CTC: chlortetracycline. The same row with different lowercase letters indicates significant differences (*p* < 0.05).

**Table 4:** Effects of Vanillin on Duodenum Morphology in Broilers.

Items	Control	CTC	0.1% Vanillin	0.2% Vanillin	0.4% Vanillin
Duodenum					
Villus height	2376.34 ± 153.03	2305.36 ± 267.57	2299.99 ± 194.12	2567.04 ± 197.83	2226.14 ± 212.64
Crypt depth	448.44 ± 53.83 ^b^	347.45 ± 13.60 ^a^	285.79 ± 40.50 ^a^	456.82 ± 101.68 ^b^	336.76 ± 50.09 ^a^
V/C	5.34 ± 0.47 ^a^	6.63 ± 0.70 ^ab^	8.19 ± 1.46 ^b^	5.92 ± 1.68 ^a^	6.67 ± 0.60 ^ab^
Mean pathological integral	0	0.337	0.337	0.337	0.667

Control: blank control; CTC: chlortetracycline. V/C: ratio of villus height-to-crypt depth; ^a,b^ Means within a variable with no common superscript differ significantly (*p* < 0.05).

## Data Availability

The original contributions presented in the study are included in the article; further inquiries can be directed to the corresponding author.
